# A Card to the Profession

**Published:** 1856

**Authors:** Jno. T. Toland

**Affiliations:** Cincinnati


### A CARD TO THE PROFESSION.

---

GENTLEMEN:—I presume there are but few among the 2,000 dentists in the West and South who do not know me more or less intimately, either by personal acquaintance, verbal conference, or correspondence—in the sale of goods, answering communications, or otherwise. But for the information of such as do not know me, or have forgotten my name, I will here state, that for nearly SEVEN YEARS I have been actively engaged in the Dental Depot, kept by J. M. Brown, during which time I have had *much to do* in every department pertaining to the "Dental Furnishing Business," supplying the profession with instruments, apparatus and materials, superintending the manufacture of instruments, selecting and arranging for different persons, to fulfill the various indications, or suit the *tastes* or necessities of *each individual;* selecting Teeth, not only to fill the space, but also of proper shade and mold to harmonize and give full expression in each case! Selecting, packing and forwarding goods to every part of the country, that all might be supplied with the articles demanded with the *least* possible *delay,* and I flatter myself that these various duties have been performed to the *satisfaction* and *benefit* both of my employer and *those who have favored us with their orders.*

The *terms of partnership* offered by Dr. Brown being such as I did not feel inclined to accept, I have therefore resolved to change my position, and try my fortunes in a business of my own; for which purpose I have visited the East and secured the agency for all the best and most popular Teeth, and made arrangements to be supplied at all times with the best Foil, Instruments, and Materials in the country. Mr. H. R. Sherwood, the celebrated Forcep and Dental Instrument maker will hereafter work exclusively for me. We will therefore be ready to manufacture, change or repair instruments, and also furnish, at the lowest Eastern prices, all instruments and materials of Eastern manufacture.

I have opened a

DENTAL DEPOT

*at No. 38 West Fourth street,*

Where I will be pleased to see my old friends, and all members of the profession. My long intimate personal acquaintance with a majority of the dentists of the Great Mississippi Valley, my SEVEN

YEARS experience in the *details* of the Furnishing Business, and my *thorough knowledge* (thus acquired) of the *tastes, necessities* and *desires* of the PROFESSION, give me UNEQUALED advantages, for supplying their wants *promptly*, and with such articles as are in conformity with the "progressive spirit of the age," and the more rapid advancement of Dental Science.

I have long felt and believed, that there is still room for many important improvements, and these it is my intention, to the best of my ability, to adopt, and supply, by keeping constantly on hand all articles kept by others, besides many not to be found elsewhere— in a Dental Depot, and all at prices which will compare *favorably* with any establishment in the United States.

For the more thorough prosecution of the business, and in order to enable *me* to devote all necessary time to the Dental Department, I have associated with me Mr. Ellwood E. Thorne, whose courteous and gentlemanly deportment, intelligence, and thorough business habits will, I feel assured, elicit the friendship and command the respect of all with whom he may meet.

With much esteem, I am as ever,

Yours, truly,

JNO. T. TOLAND.

CINCINNATI, February 18, 1856.

N. B.—I expect to make it the *interest* of all to purchase here. "A fair field and no favor" is all I ask, and having that, the profession will soon learn where to go to get the worth of their money.

